# Mass spectrometry and bioinformatics analysis data

**DOI:** 10.1016/j.dib.2014.11.002

**Published:** 2014-11-13

**Authors:** Mainak Dutta, Elavarasan Subramani, Khushman Taunk, Akshada Gajbhiye, Shubhendu Seal, Namita Pendharkar, Snigdha Dhali, Chaitali Datta Ray, Indrani Lodh, Baidyanath Chakravarty, Swagata Dasgupta, Srikanth Rapole, Koel Chaudhury

**Affiliations:** a School of Medical Science and Technology, Indian Institute of Technology, Kharagpur, West Bengal, India; b Proteomics Lab, National Centre for Cell Science, Ganeshkhind, Pune, Maharashtra, India; c Wipro GE Healthcare, Mumbai, India; d Institute of Post Graduate Medical Education & Research, Obstetrics & Gynecology, Kolkata, West Bengal, India; e Institute of Reproductive Medicine, Sector-III, Kolkata, West Bengal, India; f Department of Chemistry, Indian Institute of Technology, Kharagpur, West Bengal, India

## Abstract

2DE and 2D-DIGE based proteomics analysis of serum from women with endometriosis revealed several proteins to be dysregulated. A complete list of these proteins along with their mass spectrometry data and subsequent bioinformatics analysis are presented here. The data is related to “Investigation of serum proteome alterations in human endometriosis” by Dutta et al. [1].

**Table:** Specifications table

Subject area	Biology
More specific subject area	Endometriosis
Type of data	Excel table, text table, figure
How data was acquired	Mass spectrometry (AB Sciex 4800 MALDI-TOF/TOF) and bioinformatics analysis was performed using PANTHER, DAVID, WebGestalt and STRING
Data format	Analyzed
Experimental factors	2DE was performed
Experimental features	Protein spots were excised and trypsin digested
Data source location	Kharagpur, India and Pune, India
Data accessibility	Data is with this article and is related to Dutta et al. [1]

## Value of the data

• Mass spectrometry data of the 25 significantly differentially expressed proteins provided.
• Molecular and biological functions of the identified proteins presented.
• Interection network generated.
• The data is expected to help in understanding of endometriosis pathophysiology and for further studies and data mining.

## 1 Data, experimental design, materials and methods

### 1.1 Sample preparation of serum

Following delipidation sample preparation was performed according to Ray et al. [2]. Briefly, the two most high abundant serum proteins (albumin and IgG) were depleted using Albumin & IgG Depletion SpinTrap (GE Healthcare) following the manufacturer׳s instructions. The depleted serum was then subjected to acetone precipitation. Four volumes of ice cold acetone were added to one volume of depleted serum following which the mixture was vortexed and incubated at −20 °C for 1 h. Next, the mixture was centrifuged at 10,000*g* for 15 min at 4 °C. The supernatant was discarded and the pellet air dried and resuspended in partial rehydration buffer (7 M urea, 2 M thiourea and 4% CHAPS). The final round of salt removal was performed using 2D Clean-up kit (GE Healthcare) following the manufacturer׳s instructions.

### 1.2 Two dimensional gel electrophoresis

Protein quantity was estimated using 2D-Quant kit (GE Healthcare) prior to 2DE. A total of 1 mg protein was diluted in Destreak rehydration buffer (GE Healthcare) and 1.2% ampholyte (pH 4–7) to make the final volume to 350 μl. Diluted samples were then subjected to passive rehydration in 18 cm IPG strips covering linear pH 4–7 gradients at room temperature. Strips were then focused on a EttanIPGphor III focusing system (GE Healthcare) at 20 °C and a maximum current of 75 μA with the following voltage gradient: 200 V for 6 h; 500 V for 2 h; 1000 V for 2 h; 8000 V (gradient) for 13,500 Vh and 8000 V for 9:30 h. Focused IPG strips were reduced with equilibration solution (6 M urea, 50 mMTris pH 8.8, 2% SDS, 1% bromophenol blue) containing 1% DTT for 15 min, followed by alkylation in equilibration solution containing 2.5% IAA for 15 min. Both incubations were performed with gentle agitation. Strips were then rinsed with Milli-Q H2O and drained on damp filter paper before second-dimension electrophoresis. PROTEAN II xi electrophoresis system (Biorad) using 12% polyacrylamide gels was used for second-dimension separation. Gels were run at 100 V for 1 h; 150 V for 2 h and 250 V for 3 h. Following electrophoresis, gels were stained in 0.1% Coomassie Brilliant Blue (CBB) stain for 1 h and destained in 40% ethanol, 10% acetic acid and 50% milliQ.

### 1.3 Difference in gel electrophoresis

Prior to DIGE experiments, pH of the serum samples was adjusted to 8.5 using 50 mMNaOH and quantified using 2D-Quant kit (GE Healthcare). 2D DIGE involves minimal labeling of epsilon amino group of lysine residues with N-hydroxysuccinimide (NHS) ester reactive groups present in CyDyes (Cy2, Cy3, and Cy5) (GE Healthcare, Germany). A pool of all 72 samples (12 samples from each stage of endometriosis and 24 controls) was labeled with Cy2 dye and used as internal standard. The depleted and desalted serum samples from ES and healthy controls were alternately labeled with Cy3 or Cy5, respectively (dye-swapping) for one experimental set. Similarly, another experimental set was performed with LS and controls samples. DIGE experiments were performed with manufacturer׳s instructions with slight modifications. Briefly, 75 μg of protein was labeled with 0.4 nmol of fluorescence CyDye (GE-Healthcare, Germany) for 1 h in ice in a total reaction volume of 17 μL. The reaction was stopped by incubating the reaction mix with 1 μL of 10 mM lysine in ice for 15 min. Lysine reacts with unreacted NHS ester present in the dyes thereby stopping the reaction. The mix was further incubated in ice for 15 min after adding 17 μL 2X sample buffer (8 M urea, 130 mM DTT, 4% CHAPS and 2% pH 4-7 ampholytes). Cy2- (internal standard), Cy3-, and Cy5-labeled samples were finally combined to form a master reaction mix of 105 μL. The volume of the mix was then made upto 350 μL using Destreak Rehydration buffer (GE Healthcare, Germany) and 1.2% pH 4–7 ampholytes. Rehydration, isoelectric focusing and SDS-PAGE were performed according to the protocol discussed previously in the two dimensional electrophoresis section. The entire DIGE experiment was performed in dark.

### 1.4 Image acquisition and software analysis

**Fig. 1:**
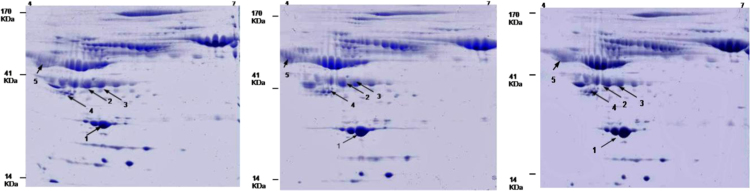
Representative image for CBB stained 2DE gel of (A) control, (B) ES and (C) LS.

After staining and destaining, the 2D gels were scanned in Image Scanner III (GE Healthcare) by using LabScan software version 6.0 (GE Healthcare) and analyzed using ImageMaster 2D Platinum 7.0 software (GE Healthcare). ES vs. controls and LS vs. controls were compared and analyzed by creating two different “match sets”. Automatic spot detection parameters were set as: Smooth: 7, Saliency: 100 and Min Area: 5. During auto detection, some non-protein spots also get detected. These contaminating artifacts including streaks and dust particles were removed by manual refinement. Percent volume calculated as (volume of the spot/total volume of all the spots detected in a gel)×100 was used for spot quantification. Representative image of 2DE gels for control, ES and LS are presented in Fig. 1.

Typhoon FLA 9500 image scanner (GE Healthcare) was used to scan the 2D DIGE gels using CyDye specific wavelengths. Subsequently, ImageQuant software; version 5.0 (GE Healthcare) was used to process the resulting gel images. Next, the gels were imported to the DeCyder 2D software; version 7.0 (GE Healthcare) for comparative spot analysis across the endometriosis and control samples. Spots were detected in the differential in-gel analysis (DIA) module of the DeCyder software, with the Decyder detection algorithm 6.0 and estimated spot number set at 2500. Gel artifacts detected as spots were manually removed. Once the spots were detected, all the gels were imported to the biological variation analysis (BVA) module of the DeCyder software for spot wise protein abundance comparison across all the experimental groups (ES vs. LS vs. controls). A statistical filter (Average ratio ≥1.5/≤−1.5; Student׳s *t*-test and one way ANOVA with *p*≤0.05) was applied to detect the most statistically significant relevant spots. False discovery rate (FDR), which takes care of the multiple testing problems, was applied to adjust the p-values obtained from ANOVA and *t*-test.

### 1.5 In-gel digestion and MALDI TOF/TOF MS

In gel trypsin digestion and mass spectrometry analysis were performed as discussed in Ray et al. [2]. Briefly, gel slices were alternatively rinsed with 25 mM ammonium bicarbonate and solution A (2:1 mixture of acetonitrile: 50 mM ammonium bicarbonate). Following repeated dehydration and rehydration, the gel slices were subjected to reduction of disulfide bonds by 10 mM DTT at 60 °C for 1 h. Alkylation step was then performed with 50 mM iodoacetamide for 20 min at room temperature in dark. Before trypsin digestion, the rehydration and dehydration steps were again performed with ammonium bicarbonate and solution A. Trypsin digestion was finally performed by incubating the gel slices overnight in 100 ng of trypsin (Trypsin Gold; Promega) at 37 °C. Following digestion, the tryptic digested fragments present in the supernatant were collected and lyophilized. Before mass spectrometry analysis, the lyophilized powder was dissolved in 0.1% TFA in 50% acetonitrile. The differentially expressed proteins were identified using AB Sciex 4800 MALDI-TOF/TOF mass spectrometer. GPS™ Explorer version 3.6 (AB Sciex) software was used for combined search of MS and MS/MS peak lists. Protein identification against the peak list was performed in MASCOT version 2.1 with Swiss-Prot database as the search engine. The search parameters for database search using Mascot were given as, taxonomy; human, enzyme used for digestion; trypsin with one missed cleavage allowed, fixed modification specified as carbamidomethylation (C) and oxidation (M) as variable modification. The peptide mass tolerance was set as 75 ppm and 0.4 Da for MS/MS tolerance. The detailed list of proteins identified using MALDI is presented in Supplementary Table 1.

### 1.6 Biological function and interaction network analysis

The differentially expressed proteins were subjected to PANTHER classification system; version 9.0 (http://www.pantherdb.org/) [3,4], DAVID and WebGestalt (http://bioinfo.vanderbilt.edu/webgestalt) for understanding biological context of the identified proteins and their involvement in biological pathways. The list of UniProt Accession number was uploaded and mapped against reference Homo sapiens dataset to extract and summarize molecular functions, biological processes and the class of proteins. The above result was further processed in order to get the proteins of the top five categories for each of the functional domains. For protein–protein interaction network analysis, the BVA analyzed differentially expressed proteins were subjected to STRING version 9.1 (http://string-db.org/) [5]. The UniProt Accession number for all the identified proteins was submitted and mapped against reference Homo sapiens dataset. The interaction networks were obtained on the basis of confidence and evidence. Predicted association between genes based on observed patterns of simultaneous expression of genes was also studied. The results of PANTHER, DAVID, WebGestalt and STRING analysis are presented in Supplementary materials 1 and 2.

### 1.7 Western blotting

40 µg protein (20 ul) of each sample was mixed with Laemmli buffer and heated at 100 °C for 10 min, then resolved in 12% sodium dodecyl sulfate-polyacrylamide gel electrophoresis (SDS-PAGE) at 120 V using running buffer. The semi-dry gel apparatus was used to electro-blot the resolved proteins onto polyvinylidene fluoride (PVDF) membrane (AmershamHybond-P, GE Healthcare, Munich, Germany) in Tris–glycine buffer at 90 V for 2 h. The membrane was transferred to blocking buffer (5% fat-free milk in TBST) for 1 h at RT. Membranes were incubated with the corresponding antibodies including haptaglobin (Santa Cruz Biotechnology), Ig kappa light chain (Ig kappa chain C region) (Santa Cruz Biotechnology), alpha-1β-glycoprotein (Santa Cruz Biotechnology) and β-actin (Santa Cruz Biotechnology) at 4 °C overnight. After washing 3 times with TBST, membranes were incubated for 1 h at RT with appropriate secondary antibody conjugated with horseradish peroxidase (Santa Cruz Biotechnology). The blots were then treated with chemiluminescent substrate and signals visualized by exposing the membrane to X-ray film (Santa Cruz). The protein expression intensity was calculated using ImageJ, Java-based image processing software (Rasband WS; NIH) and normalized against β-actin. Western blotting results are presented in Figure 7 [1].
