# Supplementary material for: Mass spectrometry and bioinformatics analysis data
Source: Data Brief. 2014 Nov 13;2:21–5. doi: 10.1016/j.dib.2014.11.002 (PMC4459760; doi:10.1016/j.dib.2014.11.002)
Supplement: Supplementary file 1 — Supplementary material [file mmc1.doc]

**PANTHER analysis of the differentially expressed proteins**

**
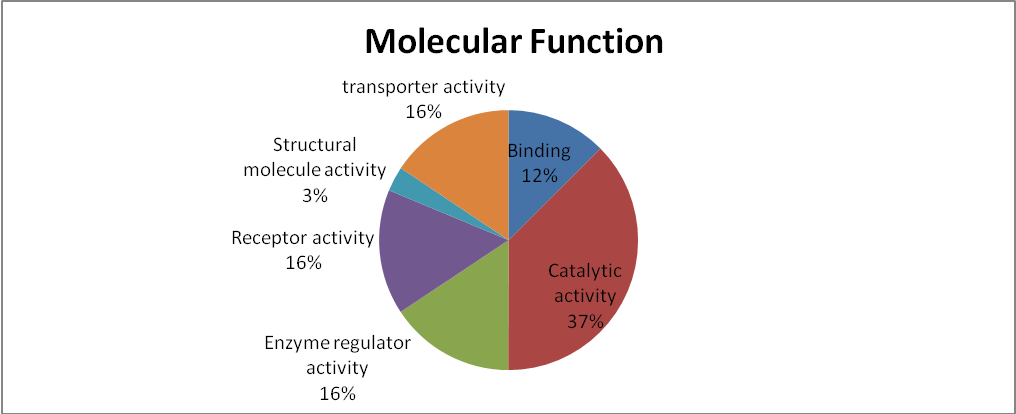
**


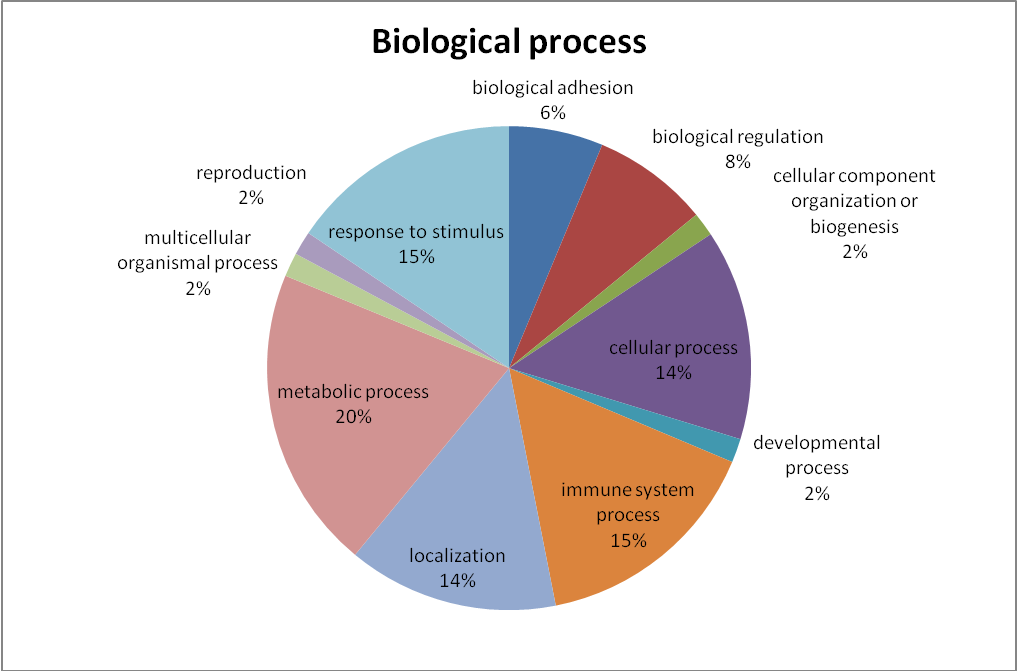
**Figure A:** Molecular Function of the identified proteins.

**Figure B:** Represents the percentage of biological processes the identified proteins are involved


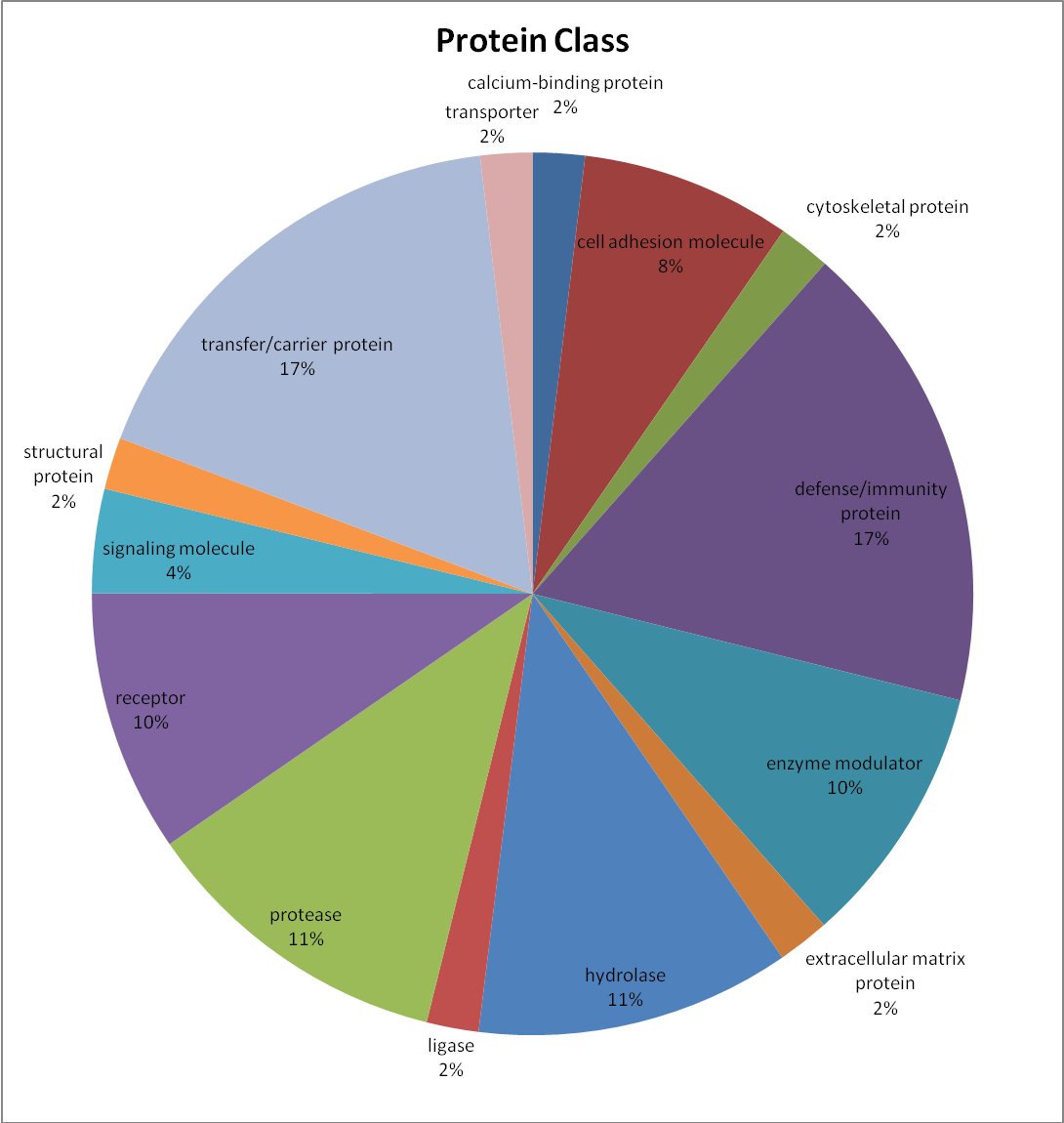


**Figure C:** Distribution of protein class of the identified proteins.

**WebGestalt analysis of the differentially expressed proteins**


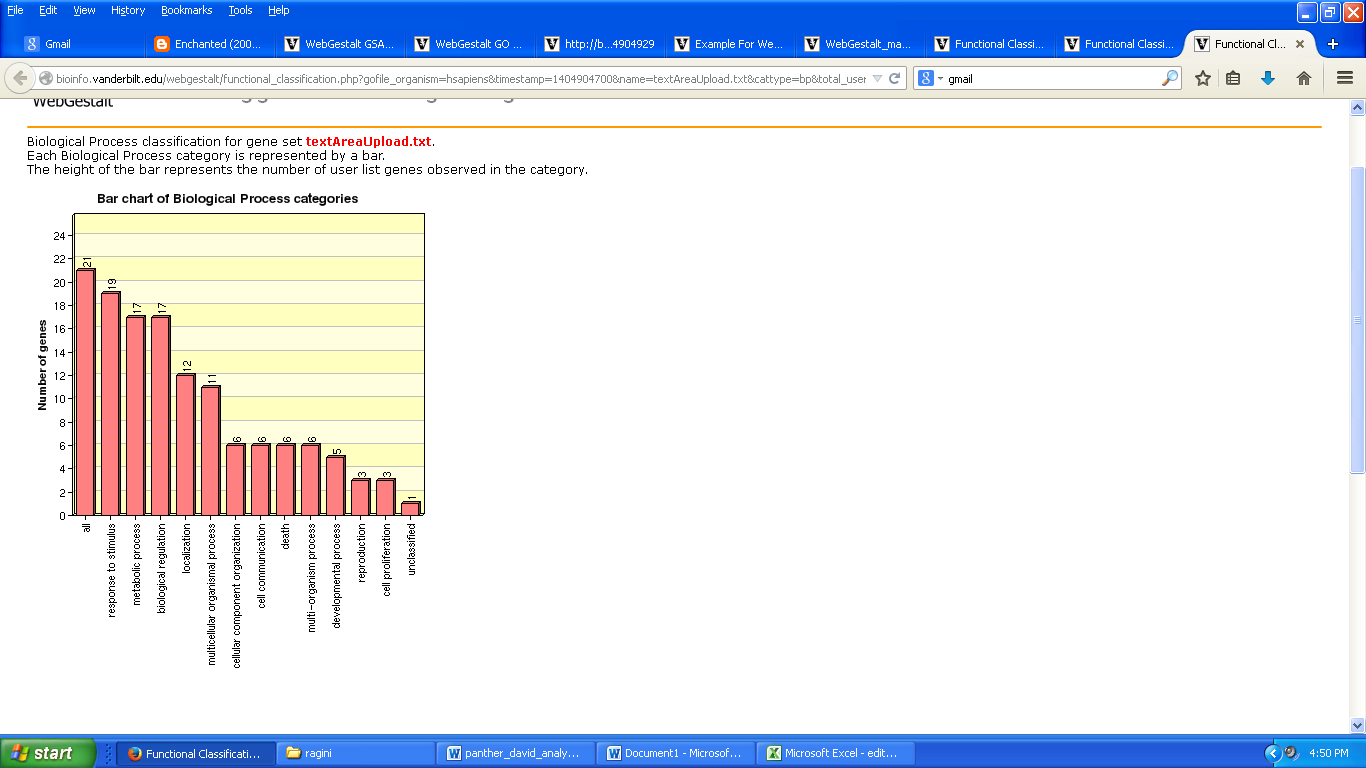


**Figure D:** Biological Process analysis


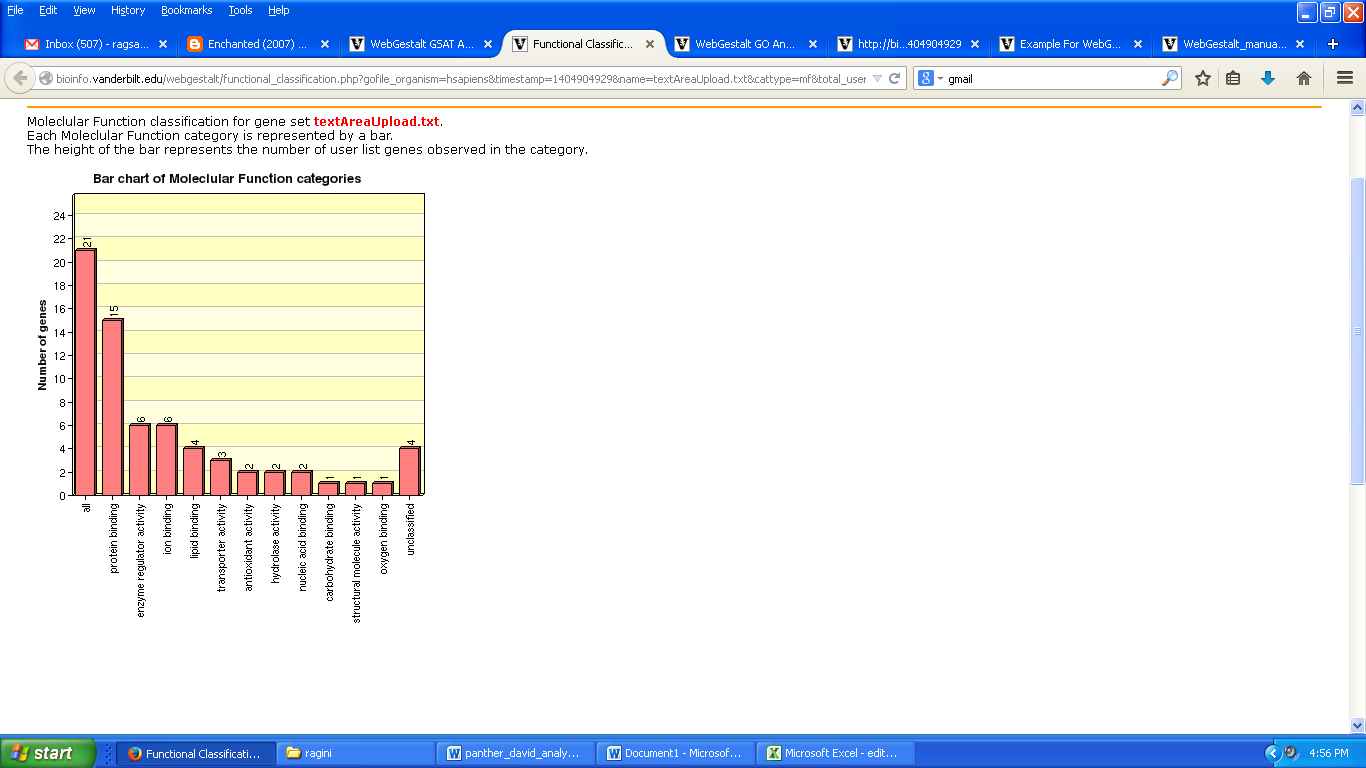
 **Figure E:** Molecular function analysis


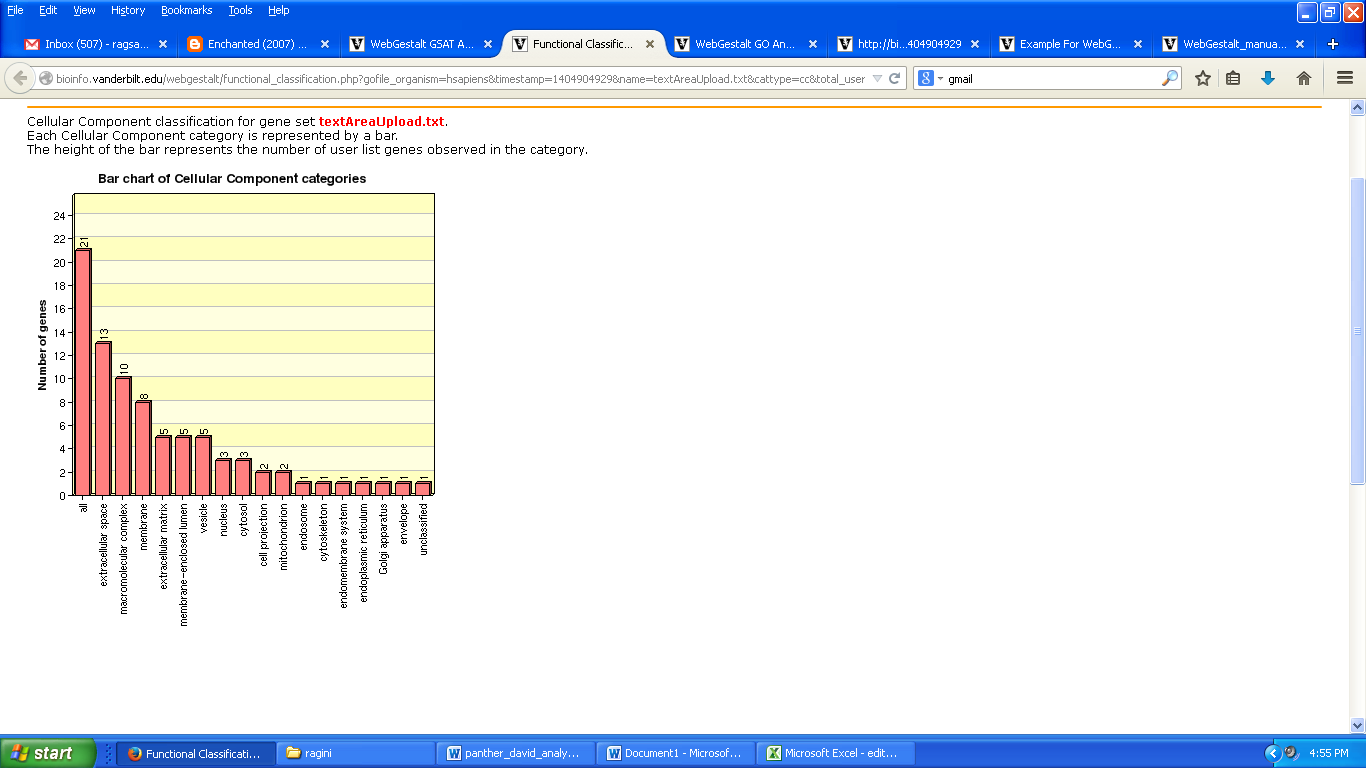


**Figure F:** Cellular component analysis

**Protein -Protein Interaction analysis using STRING**


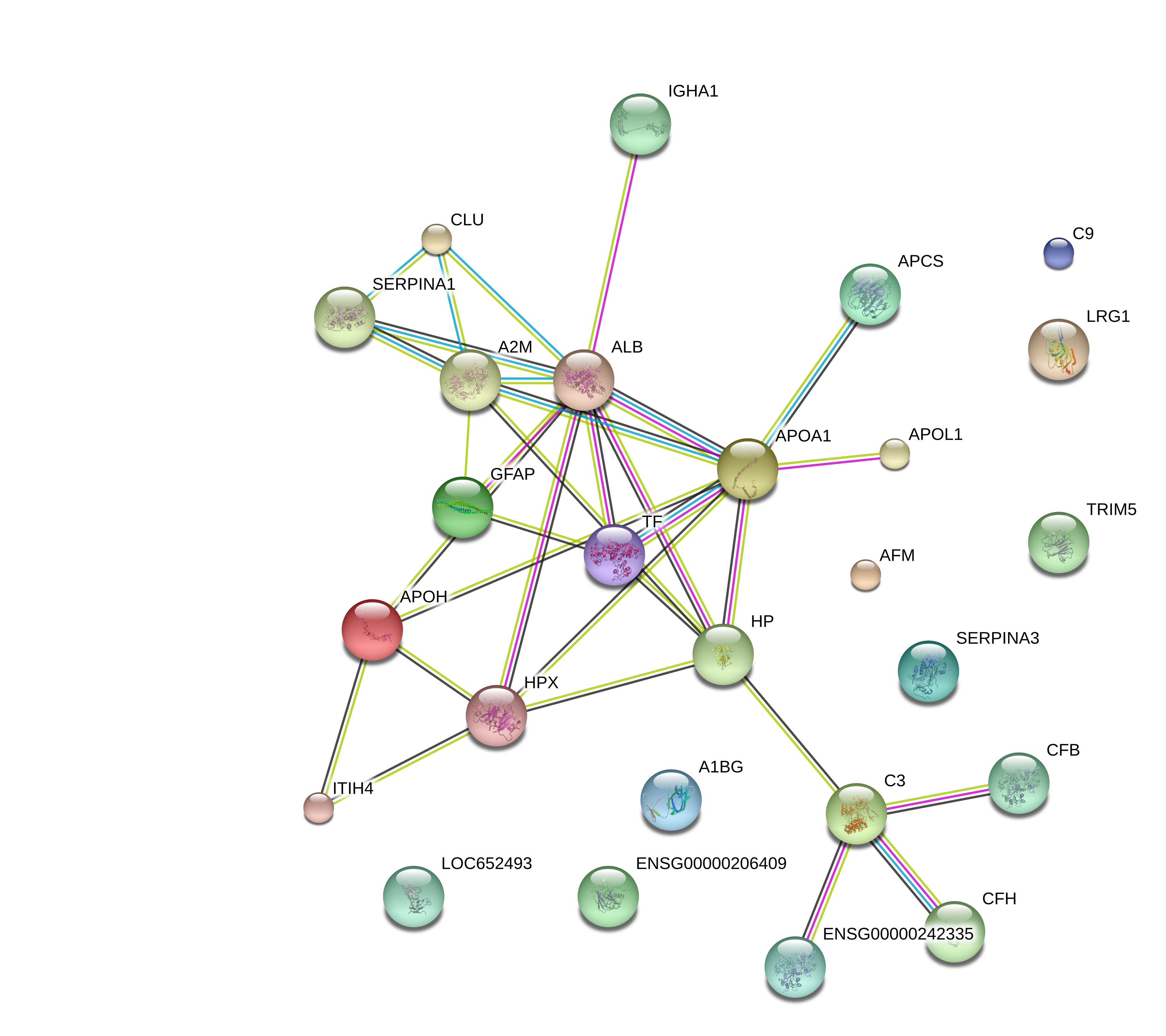


**Figure G:** The interaction networks were obtained on the basis of confidence and evidence. In the interaction network it was observed that out of the 25 proteins, 8 proteins are not showing any kind of interaction. The rest of the 17 proteins are predicted to interact with each other in some or the other pathway. In the evidence view more the number of interconnecting lines between the two proteins stronger is the evidence for the protein-protein interaction. The different colors depict different evidences of the protein-protein interaction. Predicted association between genes based on observed patterns of simultaneous expression of genes was also studied.
