# Supplementary material for: Mass spectrometry and bioinformatics analysis data
Source: Data Brief. 2014 Nov 13;2:21–5. doi: 10.1016/j.dib.2014.11.002 (PMC4459760; doi:10.1016/j.dib.2014.11.002)
Supplement: Supplementary file 2 — Supplementary material [file mmc2.doc]

Supplementary Table 1: Master list of proteins identified in MALDI TOF/TOF MS from 2DE and DIGE experiments.

| **Sl No** | **Name of protein** | **Mol. Wt. (KDa)** | **Protein score** | **Total ion score** | **No of peptides matched** | **Peptide coverage** | **Biological process^#^** | **Protein class^#^** |
| --- | --- | --- | --- | --- | --- | --- | --- | --- |
| 1. | Afamin | 70.96 | 577 | 412 | 28 | VNCLQTR  HVCGALLK  FTFEYSR  LCFFYNK  CQAYESNR  AIPVTQYLK  RPCFESLK  LPNNVLQEK  DMVEYKDR  LCFFYNKK  DADPDTFFAK  RLCFFYNK  HFQNLGKDGLK  HPDLSIPELLR  TINPAVDHCCK  RPCFESLKADK  FTDSENVCQER  RHPDLSIPELLR  ESLLNHFLYEVAR  AESPEVCFNEESPK  GQCIINSNKDDRPK  IAPQLSTEELVSLGEK  SDVGFLPPFPTLDPEEK  SCCEEQNKVNCLQTR  RNPFVFAPTLLTVAVHFE EVAK  LKHELTDEELQSLFTNFA NVVDK  ELISLVEDVSSNYDGCCE GDVVQCIR  FIEDNIEYITIIAFAQYVQE ATFEEMEK | transport | transfer/carrier protein |
| 2. | Inter-alpha-trypsin inhibitor heavy chain H4 | 103.52 | 204 | 187 | 10 | VQGNDHSATR  GWNRQAGAAGSR  AGFSWIEVTFK  AEAQAQYSAAVAK  AEAQAQYSAAVAKGK  ANTVQEATFQMELPK  NPLVWVHASPEHVVVTR  RLDYQEGPPGVEISCWS VEL  QGPVNLLSDPEQGVEVT GQYER  FSSHVGGTLGQFYQEVL WGSPAASDDGR | | [proteolysis](http://www.pantherdb.org/panther/category.do?categoryAcc=GO:0006508) [regulation of catalytic activity](http://www.pantherdb.org/panther/category.do?categoryAcc=GO:0050790) |  | | --- | --- | | [serine protease inhibitor](http://www.pantherdb.org/panther/category.do?categoryAcc=PC00204) |
| 3. | Complement factor B | 86.85 | 158 | 106 | 11 | EKLQDEDLGFL  QPWQAKISVIRPSK  DFHINLFQVLPWLK  EAGIPEFYDYDVALIK  FLCTGGVSPYADPNTCR  YGQTIRPICLPCTEGTTR  AIHCPRPHDFENGEYWP R  AIHCPRPHDFENGEYWP R  WSGQTAICDNGAGYCSN PGIPIGTR  WSGQTAICDNGAGYCSN PGIPIGTR  LLQEGQALEYVCPSGFY PYPVQTR | [complement activation](http://www.pantherdb.org/panther/category.do?categoryAcc=GO:0006956) [proteolysis](http://www.pantherdb.org/panther/category.do?categoryAcc=GO:0006508) [cell communication](http://www.pantherdb.org/panther/category.do?categoryAcc=GO:0007154) [cell-cell adhesion](http://www.pantherdb.org/panther/category.do?categoryAcc=GO:0016337) [blood coagulation](http://www.pantherdb.org/panther/category.do?categoryAcc=GO:0007596) [lipid transport](http://www.pantherdb.org/panther/category.do?categoryAcc=GO:0006869) | [apolipoprotein](http://www.pantherdb.org/panther/category.do?categoryAcc=PC00052) [receptor](http://www.pantherdb.org/panther/category.do?categoryAcc=PC00197) [metalloprotease](http://www.pantherdb.org/panther/category.do?categoryAcc=PC00153) [serine protease](http://www.pantherdb.org/panther/category.do?categoryAcc=PC00203) [metalloprotease](http://www.pantherdb.org/panther/category.do?categoryAcc=PC00153) [serine protease](http://www.pantherdb.org/panther/category.do?categoryAcc=PC00203) [complement component](http://www.pantherdb.org/panther/category.do?categoryAcc=PC00078) [cell adhesion molecule](http://www.pantherdb.org/panther/category.do?categoryAcc=PC00069) |
| 4. | Ig kappa chain C region | 11.77 | 138 | 115 | 4 | SFNRGEC  SGTASVVCLLNNFYPR  VYACEVTHQGLSSPVTK  TVAAPSVFIFPPSDEQLK | No results | No results |
| 5. | Haptoglobin | 45.86 | 476 | 413 | 14 | DYAEVGR  GSFPWQAK  QKVSVNER  VGYVSGWGR  VTSIQDWVQK  SCAVAEYGVYVK  DIAPTLTLYVGKK  VGYVSGWGRNANFK  YVMLPVADQDQCIR  YVMLPVADQDQCIR  VMPICLPSKDYAEVGR  VMPICLPSKDYAEVGR  SPVGVQPILNEHTFCAG MSK  SPVGVQPILNEHTFCAG MSK | [gamete generation](http://www.pantherdb.org/panther/category.do?categoryAcc=GO:0007276) [complement activation](http://www.pantherdb.org/panther/category.do?categoryAcc=GO:0006956) [proteolysis](http://www.pantherdb.org/panther/category.do?categoryAcc=GO:0006508) [cellular process](http://www.pantherdb.org/panther/category.do?categoryAcc=GO:0009987) [blood circulation](http://www.pantherdb.org/panther/category.do?categoryAcc=GO:0008015) [response to stress](http://www.pantherdb.org/panther/category.do?categoryAcc=GO:0006950) [blood coagulation](http://www.pantherdb.org/panther/category.do?categoryAcc=GO:0007596) | |  | [serine protease](http://www.pantherdb.org/panther/category.do?categoryAcc=PC00203) [serine protease](http://www.pantherdb.org/panther/category.do?categoryAcc=PC00203) [complement component](http://www.pantherdb.org/panther/category.do?categoryAcc=PC00078) [annexin](http://www.pantherdb.org/panther/category.do?categoryAcc=PC00050) [calmodulin](http://www.pantherdb.org/panther/category.do?categoryAcc=PC00061) | | --- | --- | |
| 6. | Tripartite motif-containing protein 5 | 57.50 | 70 | - | 12 | TQIQYDK  LQAALEMLR  HVANIVEKLR  LQAALEMLRQK  TNVLADFEQLR  ISYQPENIRPNR  LSPEGQKVDHCAR  EYQVKLQAALEMLR  TQIQYDKTNVLADFEQLR  TAWILGVCAGFQPDAMC NIEK  EEEDILKSLTNSETEMVQ QTQSLR  DILDWEESNELQNLEKEE EDILK | |  | [immune response](http://www.pantherdb.org/panther/category.do?categoryAcc=GO:0006955) [cellular protein modification process](http://www.pantherdb.org/panther/category.do?categoryAcc=GO:0006464) [response to stimulus](http://www.pantherdb.org/panther/category.do?categoryAcc=GO:0050896) | | --- | --- | | [ubiquitin-protein ligase](http://www.pantherdb.org/panther/category.do?categoryAcc=PC00234) |
| 7. | Serum albumin | 71.31 | 125 | 84 | 13 | FQNALLVR  NECFLQHK  CCTESLVNR  AWAVARLSQR  AVMDDFAAFVEK  RHPDYSVVLLLR  DVFLGMFLYEYAR  HPYFYAPELLFFAKR  RPCFSALEVDETYVPK  VFDEFKPLVEEPQNLIK  EFNAETFTFHADICTLSEK ER  QNCELFEQLGEYKFQNA LLVR  RMPCAEDYLSVVLNQLC VLHEK | transport | |  | [transfer/carrier protein](http://www.pantherdb.org/panther/category.do?categoryAcc=PC00219) |  | | --- | --- | --- | |
| 8. | Hemopexin | 52.38 | 594 | 447 | 24 | YPRDVR  SHKWDR  GEVPPRYPR  RLWWLDLK  DYFMPCPGR  DYFMPCPGR  NFPSPVDAAFR  FDPVRGEVPPR  SWPAVGNCSSALR  EWFWDLATGTMK  YYCFQGNQFLR  DVRDYFMPCPGR  WKNFPSPVDAAFR  GECQAEGVLFFQGDR  LYLVQGTQVYVFLTK  SGAQATWTELPWPHEK  LLQDEFPGIPSPLDAAVE CHR  EVGTPHGIILDSVDAAFIC PGSSR  SGAQATWTELPWPHEKV DGALCMEK  CSPHLVLSALTSDNHGAT YAFSGTHYWR  DGWHSWPIAHQWPQGP SAVDAAFSWEEK  SLGPNSCSANGPGLYLIH GPNLYCYSDVEK  **SLGPNSCSANGPGLYLIH GPNLYCYSDVEKLNAAK** | |  | [proteolysis](http://www.pantherdb.org/panther/category.do?categoryAcc=GO:0006508) [vitamin transport](http://www.pantherdb.org/panther/category.do?categoryAcc=GO:0051180) | | --- | --- | | | [transfer/carrier protein](http://www.pantherdb.org/panther/category.do?categoryAcc=PC00219) [metalloprotease](http://www.pantherdb.org/panther/category.do?categoryAcc=PC00153) [metalloprotease](http://www.pantherdb.org/panther/category.do?categoryAcc=PC00153) [extracellular matrix protein](http://www.pantherdb.org/panther/category.do?categoryAcc=PC00102) |  | | --- | --- | |
| 9 | Apolipoprotein M | 21.24 | 178 | 110 | 8 | | CVEEFK | | --- | | AFLLTPR | | FLLYNR | | MKDGLCVPR | | SPHPPEKCVEEFK | | WIYHLTEGSTDLR | | KWIYHLTEGSTDLR | | *EFPEVHLGQWYFIAGAAPTKG SAPMQLHLR* | | No hits | No hits |
| 10. | Ig alpha-1 chain C region | 38.49 | 395 | 322 | 15 | YLTWASR  SAVQGPPER  GFSPKDVLVR  EKYLTWASR  WLQGSQELPR  DASGVTFTWTPSSGK  QEPSQGTTTFAVTSILR  GDTFSCMVGHEALPLAF TQK  KGDTFSCMVGHEALPLA  DLCGCYSVSSVLPGCAE PWNHGK  KGDTFSCMVGHEALPLA FTQK  NFPPSQDASGDLYTTSS QLTLPATQCLAGK  SAVQGPPERDLCGCYSV SSVLPGCAEPWNHGK  SGNTFRPEVHLLPPPSEE LALNELVTLTCLAR  SGNTFRPEVHLLPPPSEE LALNELVTLTCLAR | No hits | No hits |
| 11. | Apolipoprotein L1 | 44.0 | 179 | 135 | 14 | NEADELRK  KVAQELEEK  LNILNNNYK  LNILNNNYK  LNILNNNYK  ALADGVQKVHK  VNEPSILEMSR  VNEPSILEMSR  NWHDKGQQYR  VNEPSILEMSRGVK  ANLQSVPHASASRPR  VTEPISAESGEQVER  EFLGENISNFLSLAGNTY QLTR  EVREFLGENISNFLSLAG NTYQLTR | [cholesterol metabolic process](http://www.pantherdb.org/panther/category.do?categoryAcc=GO:0008203) [lipid transport](http://www.pantherdb.org/panther/category.do?categoryAcc=GO:0006869) | |  | [transporter](http://www.pantherdb.org/panther/category.do?categoryAcc=PC00227) [apolipoprotein](http://www.pantherdb.org/panther/category.do?categoryAcc=PC00052) |  | | --- | --- | --- | |
| 12. | Alpha-1B-glycoprotein | 54.79 | 108 | 81 | 6 | HQFLLTGDTQGR  CEGPIPDVTFELLR  LELHVDGPPPRPQLR  VTLTCVAPLSGVDFQLR  IFFHLNAVALGDGGHYTCR  TPGAAANLELIFVGPQHA GNYR | [natural killer cell activation](http://www.pantherdb.org/panther/category.do?categoryAcc=GO:0030101) [cell-cell signaling](http://www.pantherdb.org/panther/category.do?categoryAcc=GO:0007267) [response to stimulus](http://www.pantherdb.org/panther/category.do?categoryAcc=GO:0050896) | |  | [immunoglobulin receptor superfamily](http://www.pantherdb.org/panther/category.do?categoryAcc=PC00124) [immunoglobulin receptor superfamily](http://www.pantherdb.org/panther/category.do?categoryAcc=PC00124) | | --- | --- | |
| 13. | Beta-2-glycoprotein 1 | 39.58 | 80 | 63 | 4 | VCPFAGILENGAVR  VCPFAGILENGAVR  FICPLTGLWPINTLK  CTEEGKWSPELPVCAPII CPPPSIPTFATLR | [complement activation](http://www.pantherdb.org/panther/category.do?categoryAcc=GO:0006956) [proteolysis](http://www.pantherdb.org/panther/category.do?categoryAcc=GO:0006508) [cell communication](http://www.pantherdb.org/panther/category.do?categoryAcc=GO:0007154) [cell-cell adhesion](http://www.pantherdb.org/panther/category.do?categoryAcc=GO:0016337) [blood coagulation](http://www.pantherdb.org/panther/category.do?categoryAcc=GO:0007596) [lipid transport](http://www.pantherdb.org/panther/category.do?categoryAcc=GO:0006869) | [apolipoprotein](http://www.pantherdb.org/panther/category.do?categoryAcc=PC00052) [receptor](http://www.pantherdb.org/panther/category.do?categoryAcc=PC00197) [metalloprotease](http://www.pantherdb.org/panther/category.do?categoryAcc=PC00153) [serine protease](http://www.pantherdb.org/panther/category.do?categoryAcc=PC00203) [metalloprotease](http://www.pantherdb.org/panther/category.do?categoryAcc=PC00153) [serine protease](http://www.pantherdb.org/panther/category.do?categoryAcc=PC00203) [complement component](http://www.pantherdb.org/panther/category.do?categoryAcc=PC00078) [cell adhesion molecule](http://www.pantherdb.org/panther/category.do?categoryAcc=PC00069) |
| 14. | Serum amyloid P-component | 25.48 | 68 | 42 | 5 | VGEYSLYIGR  QGYFVEAQPK  AYSLFSYNTQGR  GLRQGYFVEAQPK  IVLGQEQDSYGGKFDR | |  | [immune response](http://www.pantherdb.org/panther/category.do?categoryAcc=GO:0006955) [response to stress](http://www.pantherdb.org/panther/category.do?categoryAcc=GO:0006950) | | --- | --- | | |  | [antibacterial response protein](http://www.pantherdb.org/panther/category.do?categoryAcc=PC00051) | | --- | --- | |
| 15. | Serotransferrin | 79.29 | 711 | 500 | 33 | SCHTAVGR  APNHAVVTR  ASYLDCIR  KASYLDCIR  WCALSHHER  DSGFQMNQLR  HSTIFENLANK  EFQLFSSPHGK  EFQLFSSPHGK  EGYYGYTGAFR  KDSGFQMNQLR  DYELLCLDGTR  SASDLTWDNLKGK  CGLVPVLAENYNK  MYLGYEYVTAIR  SKEFQLFSSPHGK  MYLGYEYVTAIR  LKCDEWSVNSVGK  CSTSSLLEACTFR  DQYELLCLDNTR  DLLFRDDTVCLAK  FDEFFSEGCAPGSK  KPVEEYANCHLAR  HSTIFENLANKADR  EDPQTFYYAVAVVK  KCSTSSLLEACTFR  LCMGSGLNLCEPNNK  ADRDQYELLCLDNTR  NLNEKDYELLCLDGTR  DCHLAQVPSHTVVARSM GGK  SAGWNIPIGLLYCDLPEPR  DSSLCKLCMGSGLNLCE PNNK  KPVDEYKDCHLAQVPSH TVVAR | transport | |  | [transfer/carrier protein](http://www.pantherdb.org/panther/category.do?categoryAcc=PC00219) |  | | --- | --- | --- | |
| 16. | Alpha-1-antitrypsin | 46.7 | 166 | 116 | 15 | SVLGQLGITK  FLENEDRR  WERPFEVK  KLSSWVLLMK  LGMFNIQHCK  LGMFNIQHCK  GKWERPFEVK  LGMFNIQHCKK  RLGMFNIQHCK  SPLFMGKVVNPTQK  VFSNGADLSGVTEEAPLK  VFSNGADLSGVTEEAPLK  FNKPFVFLMIEQNTK  VFSNGADLSGVTEEAPLK LSK  **LYHSEAFTVNFGDTEEAK K** | | [proteolysis](http://www.pantherdb.org/panther/category.do?categoryAcc=GO:0006508) [regulation of biological process](http://www.pantherdb.org/panther/category.do?categoryAcc=GO:0050789) [regulation of catalytic activity](http://www.pantherdb.org/panther/category.do?categoryAcc=GO:0050790) |  | | --- | --- | | [serine protease inhibitor](http://www.pantherdb.org/panther/category.do?categoryAcc=PC00204) |
| 17. | Alpha-2-macroglobulin | 164.61 | 477 | 416 | 30 | FEVQVTVPK  QGIPFFGQVR  AIGYLNTGYQR  VGFYESDVMGR  VTAAPQSVCALR  VGFYESDVMGR  GVPIPNKVIFIR  EQAPHCICANGR  HYDGSYSTFGER  NQGNTWLTAFVLK  LVHVEEPHTETVR  IAQWQSFQLEGGLK  DNSVHWERPQKPK  TEHPFTVEEFVLPK  LLIYAVLPTGDVIGDSAK  YGRNQGNTWLTAFVLK  GHFSISIPVKSDIAPVAR  FSGQLNSHGCFYQQVK  FSGQLNSHGCFYQQVK  VDLSFSPSQSLPASHAHL R  MCPQLQQYEMHGPEGL R  AGAFCLSEDAGLGISSTA SLR  MCPQLQQYEMHGPEGL R  MCPQLQQYEMHGPEGL R  MCPQLQQYEMHGPEGL R  LHTEAQIQEEGTVVELTG R  KYSDASDCHGEDSQAFC EK  SLFTDLEAENDVLHCVAF AVPK  **QQNAQGGFSSTQDTVVA LHALSKYGAATFTR** | | [complement activation](http://www.pantherdb.org/panther/category.do?categoryAcc=GO:0006956) [proteolysis](http://www.pantherdb.org/panther/category.do?categoryAcc=GO:0006508) [cellular process](http://www.pantherdb.org/panther/category.do?categoryAcc=GO:0009987) [response to stimulus](http://www.pantherdb.org/panther/category.do?categoryAcc=GO:0050896) [regulation of catalytic activity](http://www.pantherdb.org/panther/category.do?categoryAcc=GO:0050790) |  | | --- | --- | | [cytokine](http://www.pantherdb.org/panther/category.do?categoryAcc=PC00083) [serine protease inhibitor](http://www.pantherdb.org/panther/category.do?categoryAcc=PC00204) [complement component](http://www.pantherdb.org/panther/category.do?categoryAcc=PC00078) |
| 18. | Complement factor H | 143.68 | 527 | 272 | 46 | NGFYPATR  NGFYPATR  WTGRPTCR  NGQWSEPPK  IDVHLVPDR  IIYKENER  WSHPPSCIK  EFDHNSNIR  RPYFPVAVGK  CLHPCVISR  SLGNVIMVCR  CLPVTAPENGK  LSYTCEGGFR  HGGLYHENMR  HGGLYHENMR  TGDEITYQCR  CTSTGWIPAPR  KEFDHNSNIR 1259.5682 -0.0446 -35 Mascot  497 1262.5835 507 DGWSAQPTCIK  GEWVALNPLRK  CNMGYEYSER  CNMGYEYSER  SCDIPVFMNAR  TGESVEFVCKR  CNMGYEYSER  SPDVINGSPISQK  EIMENYNIALR  SSNLIILEEHLK  MDGASNVTCINSR  CFEGFGIDGPAIAK  CTLKPCDYPDIK  SIDVACHPGYALPK  WSSPPQCEGLPCK  GKEGWIHTVCINGR  AGEQVTYTCATYYK  SCDNPYIPNGDYSPLR  SCDNPYIPNGDYSPLR  AQTTVTCMENGWSPTPR  AQTTVTCMENGWSPTPR  EQVQSCGPPPELLNGNV K  DTSCVNPPTVQNAYIVSR  IEGDEEMHCSDDGFWSK  GDAVCTESGWRPLPSCE EK  CYFPYLENGYNQNYGRK  IVSSAMEPDREYHFGQA VR  IVSSAMEPDREYHFGQA VR  TKEEYGHSEVVEYYCNP R | [complement activation](http://www.pantherdb.org/panther/category.do?categoryAcc=GO:0006956) [proteolysis](http://www.pantherdb.org/panther/category.do?categoryAcc=GO:0006508) [cell communication](http://www.pantherdb.org/panther/category.do?categoryAcc=GO:0007154) [cell-cell adhesion](http://www.pantherdb.org/panther/category.do?categoryAcc=GO:0016337) [blood coagulation](http://www.pantherdb.org/panther/category.do?categoryAcc=GO:0007596) [lipid transport](http://www.pantherdb.org/panther/category.do?categoryAcc=GO:0006869) | [apolipoprotein](http://www.pantherdb.org/panther/category.do?categoryAcc=PC00052) [receptor](http://www.pantherdb.org/panther/category.do?categoryAcc=PC00197) [metalloprotease](http://www.pantherdb.org/panther/category.do?categoryAcc=PC00153) [serine protease](http://www.pantherdb.org/panther/category.do?categoryAcc=PC00203) [metalloprotease](http://www.pantherdb.org/panther/category.do?categoryAcc=PC00153) [serine protease](http://www.pantherdb.org/panther/category.do?categoryAcc=PC00203) [complement component](http://www.pantherdb.org/panther/category.do?categoryAcc=PC00078) [cell adhesion molecule](http://www.pantherdb.org/panther/category.do?categoryAcc=PC00069) |
| 19. | Complement component C9 | 64.61 | 107 | 44 | 17 | VVEESELAR  DGNTLTYYR  FEGIACEISK  TSNFNAAISLK  LSPIYNLVPVK  DRVVEESELAR  DRDGNTLTYYR  SIEVFGQFNGKR  SIEVFGQFNGKR  AIEDYINEFSVR  DVVLTTTFVDDIK  SRSIEVFGQFNGK  TEHYEEQIEAFK  RPWNVASLIYETK  AIEDYINEFSVRK  NETYQLFLSYSSKK  NRDVVLTTTFVDDIK | [complement activation](http://www.pantherdb.org/panther/category.do?categoryAcc=GO:0006956) [proteolysis](http://www.pantherdb.org/panther/category.do?categoryAcc=GO:0006508) [cell communication](http://www.pantherdb.org/panther/category.do?categoryAcc=GO:0007154) [cell-cell adhesion](http://www.pantherdb.org/panther/category.do?categoryAcc=GO:0016337) [blood coagulation](http://www.pantherdb.org/panther/category.do?categoryAcc=GO:0007596) [lipid transport](http://www.pantherdb.org/panther/category.do?categoryAcc=GO:0006869) | [apolipoprotein](http://www.pantherdb.org/panther/category.do?categoryAcc=PC00052) [receptor](http://www.pantherdb.org/panther/category.do?categoryAcc=PC00197) [metalloprotease](http://www.pantherdb.org/panther/category.do?categoryAcc=PC00153) [serine protease](http://www.pantherdb.org/panther/category.do?categoryAcc=PC00203) [metalloprotease](http://www.pantherdb.org/panther/category.do?categoryAcc=PC00153) [serine protease](http://www.pantherdb.org/panther/category.do?categoryAcc=PC00203) [complement component](http://www.pantherdb.org/panther/category.do?categoryAcc=PC00078) [cell adhesion molecule](http://www.pantherdb.org/panther/category.do?categoryAcc=PC00069) |
| 20. | Glial Fibrillary acidic protein | 49.84 | 90 | 25 | 7 | HEANDYRR  LEAENNLAAYR  SKFADLTDAAAR  LALDIEIATYR  GTNESLERQMR  LRLDQLTANSAR  ITIPVQTFSNLQIR | c [cellular process](http://www.pantherdb.org/panther/category.do?categoryAcc=GO:0009987) [cellular component morphogenesis](http://www.pantherdb.org/panther/category.do?categoryAcc=GO:0032989) [cellular component organization](http://www.pantherdb.org/panther/category.do?categoryAcc=GO:0016043) | | [structural protein](http://www.pantherdb.org/panther/category.do?categoryAcc=PC00211) [intermediate filament](http://www.pantherdb.org/panther/category.do?categoryAcc=PC00129) |  | | --- | --- | |
| 21. | Alpha-1- antichymotrypsin | 47.62 | 189 | 75 | 15 | ADLSGITGAR  EQLSLLDR  EIGELYLPK  WRDSLEFR  ITLLSALVETR  MEEVEAMLLPETLK  EQLSLLDRFTEDAK  WEMPFDPQDTHQSR  MEEVEAMLLPETLKR  LYGSEAFATDFQDSAAAK  AVLDVFEEGTEASAATAVK  AKWEMPFDPQDTHQSR  LYGSEAFATDFQDSAAAKK  RLYGSEAFATDFQDSAAAK  GTHVDLGLASANVDFAFSLYK | | [proteolysis](http://www.pantherdb.org/panther/category.do?categoryAcc=GO:0006508) [regulation of biological process](http://www.pantherdb.org/panther/category.do?categoryAcc=GO:0050789) [regulation of catalytic activity](http://www.pantherdb.org/panther/category.do?categoryAcc=GO:0050790) |  | | --- | --- | | | [serine protease inhibitor](http://www.pantherdb.org/panther/category.do?categoryAcc=PC00204) |  | | --- | --- | |
| 22. | Clusterin | 52.46 | 174 | 153 | 8 | IDSLLENDR  RPHFFFPK  QTCMKFYAR  TLLSNLEEAKK  NPKFMETVAEK  ASSIIDELFQDR  QQTHMLDVMQDHFSR  EPQDTYHYLPFSLPHR | No hits | No Hits |
| 23. | Complement component C3 | 187.05 | 213 | 224 | 22 | | VVLVAVDK |  | | --- | --- | | LKGPLLNK |  | | VTLEERLDK |  | | LCRDELCR |  | | SDDKVTLEER |  | | NTMILEICTR |  | | ENEGFTVTAEGK |  | | ENPMRFSCQR |  | | SGSDEVQVGQQR |  | | CAEENCFIQK |  | | EVVADSVWVDVK |  | | GQGTLSVVTMYHAK |  | | AVLYNYRQNQELK |  | | VYAYYNLEESCTR |  | | VLLDGVQNLRAEDLVGK |  | | VHQYFNVELIQPGAVK |  | | SEETKENEGFTVTAEGK |  | | VTIKPAPETEKRPQDAK |  | | DSITTWEILAVSMSDKK |  | | RPQDAKNTMILEICTR |  | | VQLSNDFDEYIMAIEQTIK |  | | DTWVEHWPEEDECQDEENQK | | | [complement activation](http://www.pantherdb.org/panther/category.do?categoryAcc=GO:0006956) [proteolysis](http://www.pantherdb.org/panther/category.do?categoryAcc=GO:0006508) [cellular process](http://www.pantherdb.org/panther/category.do?categoryAcc=GO:0009987) [response to stimulus](http://www.pantherdb.org/panther/category.do?categoryAcc=GO:0050896) [regulation of catalytic activity](http://www.pantherdb.org/panther/category.do?categoryAcc=GO:0050790) | [cytokine](http://www.pantherdb.org/panther/category.do?categoryAcc=PC00083) [serine protease inhibitor](http://www.pantherdb.org/panther/category.do?categoryAcc=PC00204) [complement component](http://www.pantherdb.org/panther/category.do?categoryAcc=PC00078) |
| 24. | Leucine-rich α-2-glycoprotein  precursor | 38.15 | 605 | 321 | 11 | GPLQLER  DCQVFR  VAAGAFQGLR  ALGHLDLSGNR  DLLLPQPDLR  LARVAAGAFQGLR  ENQLEVLEVSWLHGLK  TLDLGENQLETLPPDLLR  NALTGLPPGLFQASATLDTLVLK  DGFDISGNPWICDQNLSDLYR  LQELHLSSNGLESLSPEFLRPVPQLR | [extracellular region](http://www.pantherdb.org/panther/category.do?categoryAcc=GO:0005576) [extracellular matrix](http://www.pantherdb.org/panther/category.do?categoryAcc=GO:0031012) | [receptor](http://www.pantherdb.org/panther/category.do?categoryAcc=PC00197) [extracellular matrix protein](http://www.pantherdb.org/panther/category.do?categoryAcc=PC00102) |
| 25. | Apolipoprotein A-1 precursor | 30.75 | 426 | 350 | 20 | QKVEPLR  AELQEGAR  LHELQEK  AKPALEDLR  LSPLGEEMR  QKLHELQEK  QGLLPVLESFK  DLATVYVDVLK  VQPYLDDFQK  WQEEMELYR  THLAPYSDELR  LSPLGEEMRDR  VQPYLDDFQKK  VSFLSALEEYTK  DYVSQFEGSALGK  KWQEEMELYR  VEPLRAELQEGAR  LLDNWDSVTSTFSK  DSGRDYVSQFEGSALGK  LREQLGPVTQEFWDNLEK | Blood circulation | No Hits |

^#^ The biological function and the class of proteins are obtained from PANTHER analysis
